# Trunk Laterality Judgement in Chronic Low Back Pain: Influence of Low Back Pain History, Task Complexity, and Clinical Correlates

**DOI:** 10.3390/jcm14155328

**Published:** 2025-07-28

**Authors:** Thomas Matheve, Lotte Janssens, Annick Timmermans, Nina Goossens, Lieven Danneels, Hannes Meirezonne, Michiel Brandt, Liesbet De Baets

**Affiliations:** 1 Spine, Head and Pain Research Unit Ghent, Department of Rehabilitation Sciences, Ghent University, 9000 Gent, Belgium; lieven.danneels@ugent.be (L.D.); hannes.meirezonne@ugent.be (H.M.); michiel.brandt@ugent.be (M.B.); 2 REVAL Rehabilitation Research Center, Faculty of Rehabilitation Sciences, Hasselt University, Wetenschapspark 7, 3590 Diepenbeek, Belgium; lotte.janssens@uhasselt.be (L.J.); annick.timmermans@uhasselt.be (A.T.); nina.goossens@uhasselt.be (N.G.); 3 Musculoskeletal Rehabilitation Research Group, Department of Rehabilitation Sciences, Faculty of Movement and Rehabilitation Sciences, KU Leuven, 3000 Leuven, Belgium; liesbet.debaets@kuleuven.be; 4 Pain in Motion (PAIN) Research Group, Faculty of Physical Education and Physiotherapy, Department of Physiotherapy, Human Physiology and Anatomy, Vrije Universiteit Brussel, 1000 Brussels, Belgium; 5 The Leuven Centre for Algology and Pain Management, UZ Leuven—University Hospitals Leuven, 3000 Leuven, Belgium; 6 Division of Physical Medicine and Rehabilitation, UZ Leuven—University Hospitals Leuven, 3000 Leuven, Belgium

**Keywords:** low back pain, motor imagery, left/right discrimination, fear, catastrophisation

## Abstract

**Background/Objectives**: Left/right discrimination (LRD) training is increasingly being used in the treatment of chronic low back pain (CLBP). However, it is unclear whether trunk LRD-performance is impaired in CLBP patients and whether clinical parameters are related to LRD-performance. Therefore, this cross-sectional study aimed to examine (1) whether LRD-performance differs between CLBP patients and pain-free individuals; (2) whether these differences depend on the low back pain (LBP) history in pain-free individuals; (3) if clinical factors are related to LRD-performance; (4) whether LRD-task difficulty influences these results. **Methods**: Participants included 150 pain-free persons (107 with no LBP-history; 43 with past LBP) and 150 patients with CLBP. All participants performed the LRD-task in a simple and complex condition. Outcomes were reaction time and accuracy. **Results**: CLBP patients were significantly slower (Cohen’s d = 0.47 to 0.50, *p* < 0.001) and less accurate (Cohen’s d = 0.30 to 0.55, *p* < 0.001) than pain-free individuals without LBP-history, but not compared to those with past LBP (Cohen’s d reaction time = 0.07 to 0.15, *p* = 0.55; Cohen’s d accuracy = 0.03 to 0.28, *p*-value = 0.28). All participant groups were slower and less accurate in the complex condition, but between-groups differences were independent of task difficulty. Linear mixed models showed that older age and lower education were independently associated with less accuracy. When controlling for demographics, pain intensity, disability, fear of movement, pain-related worry and pain duration were not related to LRD-performance in patients with CLBP. **Conclusions**: Patients with CLBP showed impaired trunk LRD-performance compared to pain-free persons without LBP history, but not compared to those with past LBP. When controlling for demographics, clinical parameters were not related to LRD-performance in patients with CLBP. Our findings indicate that LRD-performance may remain impaired after recovering from LBP.

## 1. Introduction

Chronic low back pain (CLBP) is a disabling and multidimensional problem [1]. Besides extensive peripheral changes (e.g., in back muscle structure) [2,3], structural and functional brain alterations (e.g., cortical reorganisation) are present in patients with CLBP [4,5], which are thought to play an important role in the development and persistence of CLBP [6,7,8]. As such, treatments aiming to tackle these neuroplastic changes in the brain have gained considerable attention [9,10,11,12]. One such approach that is increasingly being integrated into CLBP rehabilitation is graded motor imagery [9,12,13], which involves the mental rehearsal of movements without performing the actual movement.

Graded motor imagery interventions typically consist of multiple phases, including left/right discrimination (LRD) training as a starting point [14,15]. During a left/right discrimination task (LRDT) of the trunk, people have to indicate as quickly and as accurately as possible to which side a person’s trunk is rotated on pictures displaying various activities. During an LRDT, we first make an initial automatic judgement, after which we mentally move our own body into the same position to confirm this initial judgement [16]. While LRDT performance is typically worse in patients with chronic upper or lower limb pain (e.g., complex regional pain syndrome) when compared to pain-free persons [17,18,19,20,21] the findings are inconsistent for patients with CLBP [17,22,23,24,25].

Left/right discrimination shows important similarities to actual movement. For example, certain brain areas (e.g., in the premotor cortex) are activated during both a LRDT and actual movement [14,26], and the time it takes to complete a LRDT is strongly correlated to the time required to perform the actual movement [16]. In this respect, it is noteworthy that actual movement behaviour is highly variable in the CLBP population [27]. This variability can partly be explained by various clinical parameters, such as LBP intensity or pain-related beliefs. For example, patients with CLBP and elevated fear of movement move slower and with less range of motion in their lower back when compared to pain-free persons [28,29,30]. Given the similarities between imagined and actual movement behaviour, it can be hypothesised that LRDT performance may also be associated with these clinical parameters. Three studies in CLBP have previously investigated this and showed mixed results [23,24,31]. However, sample sizes were small—limiting the potential for finding significant associations—and none of these studies investigated the relationships with pain-related beliefs. Moreover, one study used a LRDT containing only pictures of simple trunk movements [23], whereas clinical parameters are typically more strongly related to complex movements [28,32].

Differences in LRDT-performance between patients with CLBP and pain-free persons may also depend on the LBP history in the pain-free controls. It has been hypothesised that LRDT accuracy depends on adequate cortical proprioceptive representation of the respective body part [33,34]. Impairments in sensorimotor control that are typically present during acute LBP, do not always spontaneously resolve when the pain subsides [35]. Since proprioception is intrinsically related to sensorimotor control [36], it may be hypothesised that differences in LRDT-performance between CLBP and pain-free participants are thus contingent upon the LBP-history in the pain-free group.

Therefore, to better understand the variability in LRDT performance in CLBP and to gain more insights in potential differences between pain-free persons and patients with CLBP, the main aims of this study were to investigate: (1) whether LRDT-performance differed between pain-free persons and patients with CLBP; (2) whether these differences depended on the LBP-history in pain-free persons; and (3) whether clinical parameters were associated with LRDT-performance in patients with CLBP; and (4) whether between-group differences and associations depended on LRDT-difficulty.

## 2. Materials and Methods

This study was approved by The Ethics Committees of Hasselt University and Jessa Hospital, Belgium (B243201423040; approval date 18 November 2015). All participants provided written informed consent before being included in the study. No patients or members of the public were involved in the study’s conceptualisation and design.

### 2.1. Study Design

This is a cross-sectional study including patients with CLBP and pain-free participants that were matched for sex and age, using an age-bracket of ±2 years.

### 2.2. Participants and Recruitment

Recruitment and data collection were performed between 12 December 2016 and 15 January 2020. Patients with CLBP who were consulting a healthcare practitioner for their LBP were recruited at the department of physical medicine and rehabilitation of Jessa Hospital (Hasselt, Belgium), and via private physiotherapy and general practitioner practices. Patients with CLBP were included and tested before they started their rehabilitation in the hospital or in private physiotherapy practice. Pain-free participants were recruited via social media, flyers in public places and word of mouth.

Common inclusion criteria for both participant groups were being of an age between 18 and 65 years and having sufficient knowledge of the Dutch language. Additional inclusion criteria for patients with LBP were a diagnosis of chronic non-specific LBP (i.e., LBP > 3 months and ≥3 days/week) confirmed by a medical doctor. The term non-specific indicates that CLBP cannot be attributed to a specific pathoanatomic cause or underlying disease, which is the case for about 90% of the CLBP population [37]. Common exclusion criteria for both participant groups were being pregnant, a history of spinal surgery, experience with motor imagery training (including left/right judgement) and having performed any type of sensorimotor control training for the lumbar spine in the past year. Patients with nerve root involvement, a serious health condition (e.g., stroke) or a specific cause of LBP (e.g., axial spondyloarthritis) were excluded. Pain-free participants were also excluded when they experienced self-reported LBP in the past year.

### 2.3. Procedures

To avoid distraction, data were collected in a quiet room at the hospital or at the university, with only the participant and a research assistant present. First, participants completed self-reported measures regarding demographic and clinical parameters, after which they performed the LRDTs.

#### 2.3.1. Self-Report Measures

##### Sociodemographic Data

We collected age, sex (assigned at birth) and educational level. Participants who only completed primary or secondary education were classified as lower educated, whereas participants with at least a bachelor’s degree or who were studying for a bachelor’s degree (or higher) at the time of study participation were classified as higher educated.

##### Clinical Parameters

Pain-free persons

Pain-free persons were asked whether they had experienced an episode of LBP that interfered with daily activities that occurred more than one year ago (yes/no; retrospective assessment). Consequently, there were three groups of pain-free (PF) persons: The total group of pain-free persons irrespective of LBP history (PF-total group); pain-free persons who had never experienced disabling LBP in their lifetime (PF-noLBP group); pain-free persons who did experience disabling LBP more than one year ago (PF-LBP group).

Patients with CLBP

To assess *LBP duration*, participants with CLBP were asked to indicate the onset of their first episode of LBP and the onset of the current LBP episode. To evaluate *LBP location*, participants indicated the location of their LBP on a body chart. Locations were categorised as left, right, central or bilateral LBP. The research assistant verified the correct categorisation of the LBP location with the participants. *Pain intensity* was assessed using the Numeric Pain Rating Scale (NPRS) [38]. Participants were asked to rate their average LBP intensity over the past seven days and their current LBP intensity on a 11-point NPRS (0 = no pain; 10 = worst imaginable pain). *Disability* was evaluated using the Roland Morris Disability Questionnaire (RMDQ) [39]. The RMDQ consists of 24 questions about the effect of LBP on daily activities. Questions must be answered with a yes or no, and a higher score (range 0–24) represents a higher level of disability. *Pain-related worry* was assessed with the Pain Catastrophising Scale (PCS) [40]. The PCS contains 13 questions relating to patients’ negative thoughts and feelings during pain. Each question must be answered on a 5-point scale (0 = not at all, 4 = always), resulting in a score between 0 and 52. A higher score corresponds with a higher level of pain-related worry. *Fear of movement* was evaluated with the Tampa Scale for Kinesiophobia (TSK) [41]. The TSK contains 17 items to assess subjective ratings of fear of movement/reinjury due to physical activity. The TSK-score ranges between 17 and 68, with a higher score indicating a higher fear of movement level.

#### 2.3.2. Left/Right Discrimination Task

The commercially available Recognise^®^ App (Version 1.1.3) on a mobile tablet (iPad Mini 3, Apple, Cupertino, CA, USA) was used to assess left/right discrimination. During this task, participants were shown pictures of people who were performing activities with their trunk rotated or bent either to the left or right. In line with the originally described test procedures [14], participants were instructed to indicate as quickly and as accurately as possible to which side a person’s trunk was rotated or bent on these pictures. During the test, participants sat on a chair with the tablet placed directly in front of them on a table. Both forearms of the participants were resting on the table. Two arrow buttons (left and right) were displayed on the tablet, which the participants had to tap with their respective index fingers to indicate whether the person in the picture had their back rotated/bent to the left or right. Pictures were displayed for a maximum of seven seconds. When participants did not press a button in this time-frame, the next picture was automatically shown. Participants were not allowed to rotate the tablet or to rotate their heads or bodies to match the rotation of the person’s trunk shown in the picture. This was checked by the research assistant present in the room. When necessary, the research assistant reminded participants of these instructions. The assistant sat a few metres behind the participants to avoid any distraction.

Participants performed the LRDT in a simple and a complex condition. In the simple condition, pictures of simple back movements (e.g., simple sidebending) were shown against a neutral background. The complex condition contained pictures of more complex movements in a daily life context (e.g., a person dancing ballet). In both conditions, pictures could be rotated 90° or 180° (i.e., upside down). After participants indicated to have understood the test instructions, the LRDTs were performed in the following order. First, participants performed a familiarisation trial containing 10 pictures (5 to the left, 5 to the right) in the simple condition. After this familiarisation trial, the actual test in the simple condition was performed which contained 40 pictures (20 to the left, 20 to the right). Next, participants were allowed two minutes of rest. No feedback on task performance was provided. After this rest interval, participants performed a familiarisation trial in the complex condition (10 pictures; 5 left, 5 right), after which the actual test was performed (40 pictures; 20 left, 20 right).

### 2.4. Data Processing and Outcomes

Outcomes were accuracy (ACC) expressed in percentage (%) and reaction time (RT) expressed in seconds (s). A higher ACC and lower RT correspond with a better performance. The Recognise^®^ App automatically registers these outcomes and generates mean ACCs and RTs for pictures to the left and right side separately. When participants did not respond within 7 s, results from the respective picture were not included. We decided to pool the data from both sides and use average scores of the 40 pictures. This decision was made because there were no statistically significant differences between performances to the left or right in any of the participant groups (maximal difference in RT = 0.12 s (SD = 0.72 s), all *p*-values > 0.26; maximal difference in ACC = 4.2% (SD = 17.1%), all *p*-values > 0.11) (see Supplementary Table S1). Moreover, LBP location was not consistently associated with better or worse performance towards a specific side (e.g., those with right-sided LBP did not consistently perform better or worse to either the left or right side) (see Supplementary Table S2). Consequently, we had four outcomes: ACC and RT in the simple condition (ACC-simple, RT-simple) and ACC and RT in the complex condition (ACC-complex, RT-complex).

### 2.5. Statistical Analyses

Statistical analyses were performed with SAS JMP Pro version 17.0 (SAS institute, Cary, NC, USA). Before performing further statistical analyses, we removed outliers on the LRDTs using the outlier labelling rule [42]. Previous studies investigating relationships between sociodemographic parameters and LRDT performance in musculoskeletal pain populations have reported inconsistent results [25,43]. As such, after outlier removal, we first evaluated whether age, sex or educational level were associated with LRDT-performance. When correlation coefficients (i.e., for age) or between-groups differences (i.e., for sex or educational level) with *p*-values ≤ 0.1 were present, these sociodemographic parameters were taken into account in further analyses (i.e., in linear mixed models). To check for a trade-off between ACC and RT, we calculated correlation coefficients between both parameters for each participant group and LRDT-condition. A statistically significant positive correlation coefficient, showing that people who reacted slower (i.e., higher RT) had higher accuracy scores, would indicate such a trade-off.

To assess whether clinical parameters were associated with LRDT-performance in the LBP-group, and to evaluate whether this association was dependent on LRDT-condition (i.e., simple vs. complex), we first calculated zero-order correlation coefficients between the clinical parameters and each of the LRDT-outcomes (i.e., ACC and RT) in the simple and complex LRDT-condition. When the *p*-value of the zero-order correlation of at least one clinical parameter was ≤0.1, we performed a linear mixed model with the LRDT-outcome as dependent variable. The respective clinical parameters with *p*-values ≤ 0.1, LRDT-condition and their interaction terms were included together with sociodemographic variables when necessary. Participant ID was added as a random effect.

Linear mixed models were also performed to assess the differences on LRDT-performance between the complete group of pain-free persons and those with CLBP, and to evaluate whether these potential differences were dependent on LRDT-condition (simple vs. complex). Participant group (LBP vs. PF-Total), LRDT-condition and their interaction terms were entered as fixed effects, together with sociodemographic variables when necessary. Participant ID was added as a random effect. To evaluate whether between-group differences were dependent on LBP history in the pain-free group, the same analysis was repeated with the pain-free group split into those with and without LBP history. Cohen’s d effect sizes between the CLBP group and the PF-groups were calculated using the raw data group means and standard deviations. Effect sizes were interpreted as follows: 0.2 = small, 0.5 = moderate and 0.8 = large [44].

For correlational analyses, we calculated Pearson or Spearman correlation coefficients, depending on data distribution. Correlation coefficients were interpreted as small (<0.30), moderate (0.30–0.50) and large (>0.50) [44]. Assumptions for linear mixed models were satisfied and the significance level was set at α < 0.05. When linear mixed models including the three separate participant groups (i.e., LBP, PF-LBP, PF-noLBP) showed statistically significant between group differences, we performed Tukey post-hoc comparisons.

### 2.6. Sample Size

Sample size calculation was based on the main effect of group (PF-Total vs. CLBP group) on LRD-performance and the associations within the CLBP group. We used α = 0.05, power = 80% and allowed for 5% missing data (e.g., due to outliers or technical difficulties). Including 150 patients with CLBP and 150 pain-free persons would allow us to detect small to moderate main effects of group (f < 0.15) on LRD-performance, small interaction effects between group and task difficulty (f < 0.10) and small correlation coefficients with LRD-performance in the CLBP group (r < 0.25).

## 3. Results

### 3.1. Participant Characteristics

**Figure 1:**
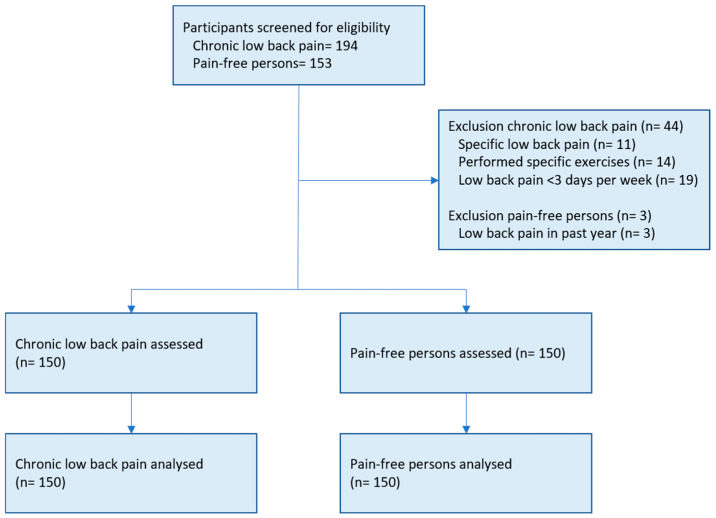
Study flowchart with reasons for exclusion.

After screening 194 patients with CLBP and 153 pain-free persons, we included 150 patients with CLBP and 150 pain-free persons that were matched for sex and age. The study flowchart with reasons for exclusion is provided in Figure 1. In the pain-free group, 107 participants had never experienced disabling LBP in their lifetime, whereas 43 participants indicated to have experienced disabling LBP more than one year before study participation. Consequently, there were four different groups: the CLBP group, the total group of pain-free persons (PF-total), a pain-free group with no LBP history (PF-noLBP) and a pain-free group with LBP history (PF-LBP). Details can be found in Table 1.

### 3.2. LRDT Outliers and Trade-Off Between Accuracy and Reaction Times

Outliers were only detected in the LRDT-simple condition. Three outliers were present in the pain-free group (one for RT; two for ACC) and two outliers were present in the CLBP group (one for RT; one for ACC). These outliers were removed from further analyses for the respective LRDT-outcomes.

For none of the participant groups (i.e., CLBP, PF-Total, PF-noLBP and PF-LBP) a trade-off between ACC and RT was found (Table 2). In contrast, seven of eight correlation coefficients were negative, indicating that participants who reacted quicker were also more accurate.

### 3.3. Associations Between Sociodemographic Variables and LRDT Outcomes

**Figure 2:**
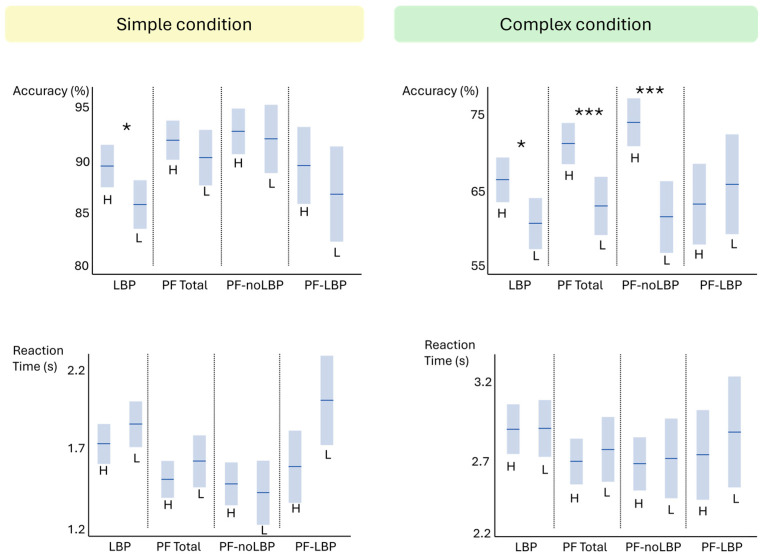
LRDT-performance for high (H) and low (L) educational level. CLBP = Chronic low back pain; PF = Pain-free; PF-LBP = Pain-free persons with a history of previous LBP; PF-noLBP = Pain-free persons without a history of previous LBP; PF-total = Total group of pain-free persons. * *p* < 0.05; *** *p* < 0.001.

For both outcomes (ACC and RT), we calculated eight correlation coefficients for the variable age (four participant groups × two conditions) (Table 3). Similarly, eight between groups differences for the variables educational level (Table 4, Figure 2) and sex (Table 5) were calculated per outcome. For ACC, six of eight correlation coefficients with age had *p*-values ≤ 0.1, whereas between groups differences for educational level had *p*-values ≤ 0.1 in four of eight comparisons. None of the between groups differences for sex had *p*-values ≤ 0.1. Therefore, we included both age and educational level in our linear mixed models for the outcome ACC. For RT, two of eight correlations coefficients with age had a *p*-value of ≤0.1, while none of the between groups differences for sex or educational level had a *p*-value ≤ 0.1. We included age in the linear mixed models for the outcome RT.

### 3.4. Differences in LRDT Between CLBP and Pain-Free Persons

**Figure 3:**
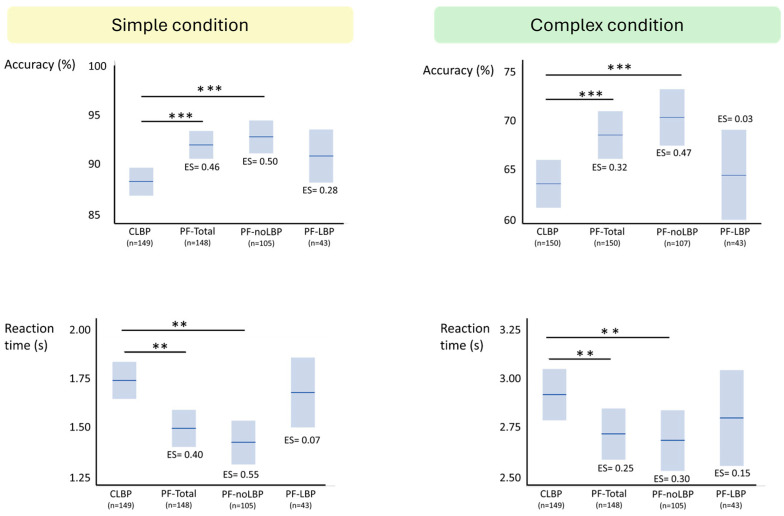
LRDT means and between-groups differences. CLBP = Chronic low back pain; PF = Pain-free; PF-LBP = Pain-free persons with a history of previous LBP; PF-noLBP = Pain-free persons without a history of previous LBP; PF-total = Total group of pain-free persons. ** Difference with CLBP group: *p* < 0.01; *** difference with CLBP group: *p* < 0.001.

Table 6 and Figure 3 provide results of the estimated means of the LRDT-performance and the effect sizes between the CLBP group and the PF-groups. Raw means are provided in Supplementary Table S3.

#### 3.4.1. Accuracy

##### CLBP vs. PF-Total Group

Main effects for participant group (*p* = 0.0003) and condition (*p* < 0.0001) were statistically significant, but not their interaction (*p* = 0.56). The CLBP-group was less accurate than the PF-total group (mean estimated difference = 3.8%, SE = 1.0%) and both groups were less accurate in the complex condition compared to the simple condition (mean estimated difference = 23.9%, SE = 0.82%).

##### CLBP vs. PF-noLBP vs. PF-LBP Groups

Significant main effects for participant group (*p* = 0.0005) and condition (*p* < 0.0001) were found, while their interaction term was not significant (*p* = 0.29). The PF-noLBP was more accurate than the CLBP group (mean estimated difference = 4.5%, SE = 1.1%, *p* = 0.0003). No statistically significant differences were present between the PF-noLBP and PF-LBP groups (mean estimated difference in favour of PF-noLBP = 2.4%, SE = 1.6%, *p* = 0.30) and between the PF-LBP and the CLBP groups (mean estimated difference in favour of PF-LBP = 2.1%, SE = 1.6%, *p* = 0.38).

#### 3.4.2. Reaction Time

##### CLBP vs. PF-Total Group

Main effects for participant group (*p* = 0.003) and condition (*p* < 0.0001) were statistically significant, while their interaction was not significant (*p* = 0.45). The CLBP-group had slower RTs compared to the PF-total group (mean estimated difference = 0.21 s, SE = 0.07 s), while both participant groups reacted slower in the complex condition than in the simple condition (mean estimated difference = 1.14 s, SE = 0.03 s).

##### CLBP vs. PF-noLBP vs. PF-LBP Groups

Main effects for participant group (*p* = 0.004) and condition (*p* < 0.0001) were statistically significant, while their interaction was not significant (*p* = 0.19). The PF-noLBP group reacted faster than the CLBP group (mean estimated difference = 0.25 s, SE = 0.07 s, *p* = 0.003), while no differences in RT were found between the PF-noLBP and PF-LBP groups (mean estimated difference in favour of PF-noLBP = 0.14 s, SE = 0.11 s, *p* = 0.40) and the PF-LBP and CLBP groups (mean estimated difference in favour PF-noLBP = 0.11 s, SE = 0.10 s, *p* = 0.55). All participant groups reacted slower in the complex than in the simple condition (mean estimated difference = 1.12 s, SE = 0.04 s).

### 3.5. Associations Between Clinical Parameters and LRDT in CLBP

**Figure 4:**
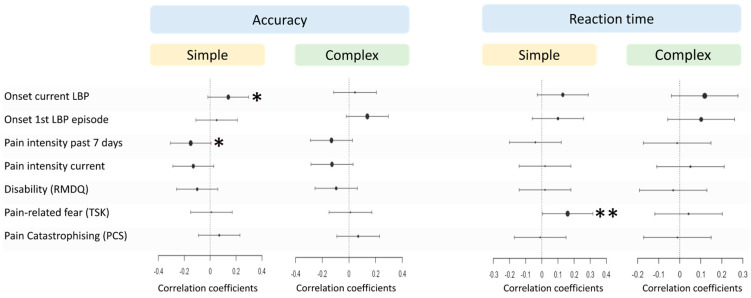
Correlation coefficients between clinical parameters and LRD-performance. * <0.1; ** <0.05.

All correlation coefficients between the clinical parameters and LRDT outcomes were small (all values r ≤ 0.16) and only three correlation coefficients had *p*-values < 0.1 (Table 7 and Figure 4). However, linear mixed models including their respective demographic variables showed that none of the main effects of the clinical parameters or their interaction with LRDT condition were statistically significant (*p* > 0.05) (Table 8). This indicates that clinical parameters were not associated with ACC and RT in both the simple and complex LRDT conditions, when controlling for demographic factors.

## 4. Discussion

We investigated LRD-performance in pain-free persons and patients with CLBP. Extending previous research, we assessed the impact of LBP-history in the pain-free control group, the effects of LRD task difficulty and the relationships between pain-related psychological factors and LRD-performance. We showed that patients with CLBP were slower and less accurate on a trunk LRDT as compared to pain-free participants. Differences between groups were contingent upon the LBP-history in the pain-free group, as patients with CLBP performed worse than pain-free participants without a lifetime history of self-reported disabling LBP, while no significant differences in LRDT-performance were found between patients with CLBP and pain-free persons with a history of disabling LBP. Significant differences with moderate effect sizes for ACC and RT were shown between patients with CLBP and pain-free persons without a history of LBP, while effect sizes were overall small and non-significant between the CLBP group and pain-free persons who had experienced LBP more than one year ago. As expected, all participant groups were slower and less accurate in the complex condition as compared to the simple condition of the LRDT. Higher age and lower educational level were associated with reduced accuracy on the LRDTs in both the CLBP and pain-free groups, although correlation coefficients with age were small. In contrast, the clinical parameters investigated in this study were not related to LRDT performance in patients with CLBP, when controlled for sociodemographic variables.

It has been hypothesised that LRDT accuracy depends on adequate cortical proprioceptive representation of the respective body part [33]. Indirect support for this hypothesis comes from Elsig et al. (2014) [34], who showed that poor performance on sensorimotor control tests (including joint repositioning error—typically considered a proxy measure of proprioception [45]) was related to less accurate LRDT performance in patients with neck pain [34]. In this respect, it is interesting that lumbopelvic sensorimotor control impairments are present in patients with recurrent LBP in remission [35,46]. Since adequate central processing of proprioceptive information is essential for sensorimotor control [36], it is possible that cortical proprioceptive representations of the lower back may be altered in pain-free participants with a history of LBP, explaining why LRDT accuracy in this participant group did not differ from participants with CLBP. However, this currently remains a hypothesis and requires further investigation, especially since participants in the PF-LBP had not experienced LBP interfering with daily life activities in the past year, while patients with recurrent LBP typically experience one or more LBP episodes per year [47].

In contrast to previous studies—reporting no differences in RT [22,23,24]—we showed that patients with CLBP were significantly slower than the total group of pain-free persons. Similar to accuracy, however, differences were only present with the PF-noLBP group and not with the PF-LBP group. Reaction times of pain-free groups in previous studies using the simple condition of the Recognize^®^ App were variable and ranged between 1.71 s and 2.4 s [23,24,25]. The RT of the PF-LBP group (=1.72 s) in the current study fell within the range of these RTs, while the RT of the PF-noLBP (=1.48 s) was faster. In two studies [23,24], no information was available on LBP-history of the pain-free group, so it is possible that a proportion of pain-free participants in these studies may have experienced previous LBP episodes, similar to the PF-LBP group. However, in contrast to the current study, Bowering et al. (2014) reported highly comparable RTs in pain-free persons with and without a history of LBP (respective RTs = 1.73 s and 1.71 s) [25], while the difference between the PF-LBP and PF-noLBP groups (respective RTs = 1.72 s and 1.48 s) in the current study was larger. To define an LBP-history, Bowering et al. (2014) [25] asked whether participants had ever experienced an LBP-episode for which they sought treatment from a health care professional, while an LBP-history in the current study was defined as having experienced at least one day of LBP that interfered with daily life activities. The LRDT in the current study was also performed under standardised conditions. For example, participants sat in a quiet room to avoid distraction, a research assistant checked whether instructions were clear and whether participants did not move their body to match the trunk rotation on the picture. Participants in the study of Bowering et al. (2014) [25] performed the LRDT at home, so the experimental environment was less controlled. However, it is unclear whether these methodological differences can explain different findings between both studies.

In summary, our results highlight the potential importance of carefully selecting inclusion criteria for pain-free control groups, especially with regards to LBP-history. Moreover, given the high recurrence rates of up to 69% in the first year after the resolution of an acute LBP-episode [48], future studies in patients with recurrent LBP in remission may be valuable to investigate potential underlying mechanisms of LBP-recurrence and to develop effective prevention programmes.

Overall, correlation coefficients between clinical parameters and LRDT performance in the CLBP group were small (all absolute r-values ≤ 0.16), and none of the clinical parameters remained significant in the linear mixed models. Three smaller (n ≤ 30) studies in CLBP found no significant correlation coefficients between LRDT performance and LBP duration [24] and disability [23,31], while mixed results were found for associations with pain intensity [23,24]. A potential reason for the lack of association is that we only used general self-report measures that did not question expected pain, perceived disability or pain-related beliefs regarding the specific tasks that were shown during the LRDTs. It has recently been shown that movement behaviour in patients with CLBP is better predicted by task-specific instead of general self-report measures (e.g., Tampa Scale for Kinesiophobia) [28]. For example, range of motion and movement velocity of the lower back during a lifting task is predicted by the perceived harmfulness of lifting with a bent back, but not by scores on the Tampa Scale for Kinesiophobia [28,29,49], which only questions beliefs regarding exercises or physical activity in general [50]. The potential importance of task-specific measures is supported by a study in patients with complex regional pain syndrome type 1 of the hand [51]. Reaction times on pictures of a hand-LRDT were strongly associated with the patients’ expected pain during those respective hand movements, while their overall current pain intensity was not related to those reaction times [51]. Therefore, future studies using task-specific measures may potentially reveal stronger associations between clinical parameters and LRDT-performance in patients with CLBP.

Both a higher age and lower educational level were independently associated with reduced accuracy. Overall, correlation coefficients between age and accuracy were small (range = −0.25 to 0.02). Educational level mainly affected accuracy in the complex condition. Three of four comparisons between educational levels (i.e., for the CLBP, PF-Total and PF-noLBP groups) were statistically significant in the complex condition, with moderate to large effect sizes (d = 0.42 to 0.89). While studies investigating LRD usually collect information on age, or match groups for this parameter, information on educational level is typically not obtained [23,24,25,33,52,53]. However, our results show this may have an important impact on LRDT accuracy. Since lower educated people are often overrepresented in certain chronic pain populations [54,55], this parameter should be taken into account as it may partially explain differences in LRDT-accuracy with pain-free participants.

### 4.1. Clinical Implications

Left/right discrimination training is increasingly being integrated into CLBP rehabilitation [9,12,13]. From a clinical perspective, it would make sense to improve LRD in those patients with impaired LRD-performance. Based on our study results, LRD-training may therefore also be potentially valuable patients with recurrent LBP, although this needs to be confirmed in future research. The general self-report measures investigated in this study were only weakly related to LRD-performance in patients with CLBP. As such, scores on these self-report measures may not be good indicators for patient selection for LRD-training. However, given the observational design of this study, these clinical implications should only be considered preliminary.

### 4.2. Implications for Future Research

Based on this work, various recommendations for future research can be made. First, future studies should assess LBP-history in pain-free participants. Moreover, investigating LRD-performance in patients with recurrent low back pain in remission would be valuable to investigate potential underlying mechanisms of LBP-recurrence. Second, when investigating relationships between LRD-performance and pain-related parameters (e.g., fear of movement), it may be worthwhile to use task-specific instead of general measures. Third, it would be valuable in future research to explore relationships between LRD-performance and sensorimotor control to further unravel underlying mechanisms of LRD-performance. Finally, when comparing participants groups, controlling for sociodemographic parameters is recommended as they may impact LRD-performance.

### 4.3. Study Limitations

First, we used the Recognise^®^ App that is commercially available. This did not allow us to receive scores on individual pictures—as the app only provides aggregate results—and prohibited us from investigating relationships between LRDT-performances and clinical parameters for specific tasks. On the other hand, it may increase external validity of our results, as clinicians can use the same protocol as described in the current study. Second, we hypothesised that the impact of LBP history on LRDT-accuracy in the pain-free group may be due to changes in proprioceptive cortical representation. Although impairments in lumbopelvic sensorimotor control can persist after resolution of a LBP-episode [35], it remains unclear if such impairments were present in the PF-LBP group. It would be valuable for future studies investigating LRDT-performance to also assess sensorimotor control—including proxy measures for proprioception—as this could either confirm or refute this hypothesis. Finally, the PF-noLBP group was clearly larger than the PF-LBP group, which we did not expect given the high lifetime prevalence and recurrence rates of LBP [48,56]. Obviously, recall bias cannot be excluded when collecting self-reported history of LBP, which may have influenced these results. The smaller sample size in the PF-LBP group reduced the power to detect statistically significant differences. Potentially, this may explain why we did not find such differences between the PF-LBP and PF-noLBP, despite moderate between group effect sizes for ACC in the complex condition and RT in the simple condition (both d = 0.43).

## 5. Conclusions

Patients with CLBP were slower and less accurate on a trunk LRDT than pain-free participants. Differences were dependent on the LBP-history in the pain-free group, as patients with CLBP performed worse than the pain-free group without a history of LBP, while no differences were present with pain-free participants with an LBP-history. When controlling for demographic variables, none of the clinical parameters were related to LRDT performance in the CLBP group. Future research in patients with recurrent LBP in remission may be worthwhile to explore potential underlying mechanisms of LBP-recurrence and to develop effective prevention programmes.

## Figures and Tables

**Table 1:** Participant characteristics.

	CLBP	Pain-Free			CLBP vs. PF-Total	CLBP vs. PF-noLBP vs. PF-LBP
		PF-Total	PF-noLBP	PF-LBP		
	Mean (SD)	Mean (SD)	Mean (SD)	Mean (SD)		
N	150	150	107	43		
Sex (n F, %F)	82 (55%)	82 (55%)	59 (55%)	23 (53%)	1.00	0.98
Age (years)	41.3 (12.5)	41.2 (12.4)	39.5 (11.9)	45.5 (12.5)	0.87	0.03 ^a^
BMI (kg/m^2^)	24.8 (3.4)	23.6 (2.7)	23.5 (2.8)	23.9 (2.1)	0.004	0.02 ^b^
Education (*n* high, %high)	85 (57%)	100 (67%)	74 (69%)	26 (60%)	0.08	0.12
Onset current LBP (years)	6.6 (8.8)					
Onset first LBP (years)	12.0 (10.4)					
LBP location (*n*, %)						
Right side	19 (13%)					
Left side	16 (11%)					
Central	62 (41%)					
Bilateral	53 (35%)					
LBP intensity 7 days (0–10)	5.0 (1.7)					
LBP intensity current (0–10)	4.1 (2.0)					
RMDQ (0–24)	8.9 (4.7)					
TSK (17–68)	36.4 (7.4)					
PCS (0–52)	18.6 (9.6)					

Data are mean (SD) unless otherwise specified. BMI = Body Mass Index; CLBP = Chronic low back pain; LBP = low back pain; PCS = Pain Catastrophising Scale; PF = Pain-free; PF-LBP = Pain-free persons with a history of previous LBP; PF-noLBP = Pain-free persons without a history of previous LBP; PF-total = Total group of pain-free persons; RMDQ = Roland Morris Disability Questionnaire; TSK = Tampa Scale for Kinesiophobia. ^a^ PF-LBP vs. PF-noLBP; ^b^ CLBP vs. PF-noLBP

**Table 2:** Correlation coefficients between accuracy and reaction times.

	CLBP (*n* = 150)	Pain-Free Persons		
		PF-Total (*n* = 150)	PF-noLBP (*n* = 107)	PF-LBP (*n* = 43)
Simple condition	−0.38 **	−0.32 **	−0.34 **	−0.27
Complex condition	−0.21 *	−0.13	−0.20 *	0.11

* *p* < 0.05; ** *p* < 0.001. Negative correlation coefficients indicate that participants who had quicker reaction times were also more accurate (= no trade-off between accuracy and reaction time). CLBP = Chronic low back pain; PF = Pain-free; PF-LBP = Pain-free persons with a history of previous LBP; PF-noLBP = Pain-free persons without a history of previous LBP; PF-total = Total group of pain-free persons.

**Table 3:** Correlations coefficients between age and LRDT outcomes.

	CLBP (*n* = 150)		PF-Total (*n* = 150)		PF-noLBP (*n* = 107)		PF-LBP (*n* = 43)	
	r	*p*	r	*p*	r	*p*	r	*p*
Accuracy (%)								
Simple	−0.22	0.008	−0.22	0.007	−0.25	0.01	−0.09	0.58
Complex	−0.14	0.08	−0.24	0.003	−0.24	0.01	0.02	0.86
Reaction Time (s)								
Simple	0.19	0.02	0.06	0.50	−0.24	0.01	−0.09	0.55
Complex	0.09	0.27	−0.04	0.66	−0.03	0.72	−0.09	0.55

CLBP = Chronic low back pain; PF = Pain-free; PF-LBP = Pain-free persons with a history of previous LBP; PF-noLBP = Pain-free persons without a history of previous LBP; PF-total = Total group of pain-free persons.

**Table 4:** Differences in LRDT performance between participants with high and low educational level.

	High Educational Level			Low Educational Level			ES (d)	*p*
	N	M	SD	N	M	SD		
**Accuracy (%)**								
*Simple*								
CLBP	85	90.1	8.6	65	86.4	10.3	0.39	0.02
PF-total	98	92.4	6.6	50	91.8	6.9	0.09	0.61
PF-noLBP	74	92.1	6.4	33	92.8	6.0	−0.11	0.60
PF-LBP	24	91.0	8.2	17	91.0	8.1	0	0.99
*Complex*								
CLBP	85	66.5	14.8	65	60.7	12.8	0.42	0.01
PF-total	100	71.2	13.9	50	63.1	13.6	0.59	0.0009
PF-noLBP	74	74.0	12.9	33	61.6	14.8	0.89	<0.0001
PF-LBP	26	63.3	13.9	17	65.9	10.8	−0.21	0.49
**Reaction time (s)**								
*Simple*								
CLBP	84	1.73	0.56	64	1.82	0.58	0.16	0.39
PF-total	100	1.52	0.54	49	1.59	0.60	0.12	0.47
PF-noLBP	74	1.49	0.54	33	1.43	0.45	−0.12	0.58
PF-LBP	26	1.60	0.55	16	1.91	0.75	0.47	0.16
*Complex*								
CLBP	85	2.89	0.80	65	2.90	0.69	0.01	0.97
PF-total	100	2.69	0.74	50	2.77	0.67	0.11	0.53
PF-noLBP	74	2.68	0.64	33	2.71	0.64	0.05	0.81
PF-LBP	26	2.73	0.59	17	2.88	0.74	0.22	0.51

CLBP = Chronic low back pain; PF = Pain-free; PF-LBP = Pain-free persons with a history of previous LBP; PF-noLBP = Pain-free persons without a history of previous LBP; PF-total = Total group of pain-free persons. Cohen’s d effect size (ES): positive values in favour of the highly educated group.

**Table 5:** LRDT accuracy and reaction times categorised per sex.

		CLBP (*n* = 150)		PF-Total (*n* = 150)		PF-noLBP (*n* = 107)		PF-LBP (*n* = 43)		Smallest *p*-Value
		M (45%)	F (55%)	M (45%)	F (55%)	M (45%)	F (55%)	M (47%)	F (53%)	
Accuracy (%)										
Simple		88.8 (8.3)	87.8 (11.1)	92.8 (5.7)	91.6 (7.4)	93.0 (5.6)	92.3 (6.6)	92.4 (6.2)	89.7 (9.2)	0.23
Complex		66.3 (14.0)	62.1 (14.2)	68.0 (14.2)	68.9 (14.6)	68.9 (15.5)	71.2 (14.0)	65.9 (10.4)	62.9 (14.5)	0.11
Reaction time (s)										
Simple		1.78 (0.61)	1.77 (0.54)	1.48 (0.52)	1.59 (0.59)	1.42 (0.47)	1.52 (0.54)	1.62 (0.61)	1.80 (0.68)	0.30
Complex		2.93 (0.81)	2.87 (0.71)	2.68 (0.70)	2.75 (0.73)	2.33 (0.73)	2.71 (0.75)	2.71 (0.63)	2.85 (0.67)	0.50

Data are mean differences between pictures to the left and right in absolute values. CLBP = Chronic low back pain; PF = Pain-free; PF-LBP = Pain-free persons with a history of previous LBP; PF-noLBP = Pain-free persons without a history of previous LBP; PF-total = Total group of pain-free persons. The smallest *p*-values of the differences between participants with male (M) and female (F) sex are provided.

**Table 6:** Estimated means for LRDT performance in the participant groups.

	CLBP (*n* = 150)		Pain-Free								
			PF-Total (*n* = 150)			PF-noLBP (*n* = 107)			PF-LBP (*n* = 43)		
	M	SE	M	SE	ES	M	SE	ES	M	SE	ES
Accuracy (%)											
Simple	88.0	0.9	91.3	0.9	0.46 ***	91.5	1.1	0.50 ***	90.9	1.8	0.28
Complex	63.5	0.9	67.9	0.9	0.32 ***	69.1	1.1	0.47 ***	64.7	1.8	0.03
Reaction time (s)											
Simple	1.77	0.05	1.54	0.05	0.40 **	1.48	0.06	0.55 **	1.72	0.10	0.07
Complex	2.90	0.05	2.71	0.05	0.25 **	2.69	0.06	0.30 **	2.75	0.10	0.15

ES = Cohen’s d effect size based on raw means and standard deviation. Effect sizes are calculated between the CLBP and respective PF groups. CLBP = Chronic low back pain; PF = Pain-free; PF-LBP = Pain-free persons with a history of previous LBP; PF-noLBP = Pain-free persons without a history of previous LBP; PF-total = total group of pain-free persons. ** Difference with CLBP group: *p* < 0.01; *** difference with CLBP group: *p* < 0.001.

**Table 7:** Zero-order correlation coefficients between clinical parameters and LRDT-outcomes in the CLBP group.

	NPRS 7D	NPRS Current	RMDQ	TSK	PCS	LBP Onset 1st Episode	LBP Onset Current Episode
Accuracy							
Simple	−0.15 *	−0.13	−0.10	0.01	0.07	0.05	0.14 *
Complex	−0.12	−0.12	−0.11	0.01	0.06	0.13	0.05
Reaction time							
Simple	−0.04	0.02	0.02	0.16 **	−0.003	0.10	0.13
Complex	−0.03	0.04	−0.01	0.04	−0.01	0.11	0.11

LBP = Low back pain; NPRS = Numeric Pain Rating Scale; NPRS current = Current pain intensity measured with NPRS; NPRS 7D = Average pain intensity over the past 7 days measured with NPRS, RMDQ = Roland Morris Disability Questionnaire; PCS = Pain Catastrophising Scale; TSK = Tampa Scale for Kinesiophobia. * *p* < 0.1; ** *p* < 0.05.

**Table 8:** Linear mixed models for associations between clinical parameters and LRDT-outcomes in the CLBP group.

	Fixed Effects	Estimate	SE	t Ratio	*p*
**Accuracy**	Intercept	86.03	3.66	23.53	<0.0001
	Age	−0.14	0.07	−2.10	0.04
	Education (Low)	−2.14	0.82	−2.62	0.009
	Condition (Complex)	−12.29	0.56	−21.77	<0.0001
	NPRS 7D	−0.90	0.49	−1.85	0.07
	NPRS 7D*Condition	−0.11	0.34	−0.32	0.75
	Onset current LBP	−0.001	0.10	−0.02	0.99
	Onset current LBP*Condition	0.05	0.07	0.78	0.43
**Reaction time**	Intercept	1.85	0.28	6.64	<0.001
	Age	0.006	0.004	1.60	0.11
	Condition (Complex)	0.56	0.02	22.75	<0.001
	TSK	0.006	0.007	0.95	0.35
	TSK*Condition	−0.004	0.003	−1.22	0.23

LBP = Low back pain; NPRS 7D = Numeric pain rating scale for pain intensity in the past 7 days; TSK = Tampa Scale for Kinesiophobia.

## Data Availability

The raw data supporting the conclusions of this article will be made available by the authors on request.

## Supplementary Materials

The following supporting information can be downloaded at: https://www.mdpi.com/article/10.3390/jcm14155328/s1, Figure S1: Study flowchart; Table S1: Differences in accuracy and reaction time between pictures to the left and right; Table S2: LRDT performances in the CLBP-group based on LBP location; Table S3: Raw means for LRDT performance in the different participant groups.

## Abbreviations

The following abbreviations are used in this manuscript:

ACC	Accuracy
CLBP	Chronic low back pain
LBP	Low back pain
LRD	Left/right discrimination
LRDT	Left/right discrimination task
PF	Pain-free
PF-LBP	Pain-free with low back pain history
PF-noLBP	Pain-free without low back pain history
RT	Reaction time
