# Supplementary material for: Trunk Laterality Judgement in Chronic Low Back Pain: Influence of Low Back Pain History, Task Complexity, and Clinical Correlates
Source: J Clin Med. 2025 Jul 28;14(15):5328. doi: 10.3390/jcm14155328 (PMC12347674; doi:10.3390/jcm14155328)
Supplement: Supplementary file 1 [file jcm-14-05328-s001.zip › Table S1.pdf]

Supplementary Table S1. Differences in accuracy and reaction time between pictures to the left and right

	CLBP (N=150)		PF-total (N=150)		PF-noLBP (N=107)		PF-LBP (N=43)		Smallest p-value
	M	SD	M	SD	M	SD	M	SD	
Accuracy (%)									
Simple	0.8	9.8	1.0	8.6	1.1	8.3	0.8	8.5	> 0.16
Complex	0.6	14.7	0.5	14.7	0.9	14.5	4.2	17.1	> 0.11
Reaction time (s)									
Simple	0.04	0.50	0.02	0.37	0.04	0.41	0.03	0.39	> 0.26
Complex	0.04	0.86	0.002	0.73	-0.04	0.69	0.12	0.72	> 0.28

Data are mean differences between pictures to the left and right in absolute values
CLBP= chronic low back pain; PF= Pain-free; PF-LBP= Pain-free persons with a history of previous LBP; PF-noLBP= pain-free persons without a history of previous LBP; PF-total= total group of pain-free persons
The smallest p-value of the between-groups comparisons per outcome is shown.
