# Supplementary material for: Trunk Laterality Judgement in Chronic Low Back Pain: Influence of Low Back Pain History, Task Complexity, and Clinical Correlates
Source: J Clin Med. 2025 Jul 28;14(15):5328. doi: 10.3390/jcm14155328 (PMC12347674; doi:10.3390/jcm14155328)
Supplement: Supplementary file 1 [file jcm-14-05328-s001.zip › Table S2.pdf]

Supplementary Table S2. LRDT performances in the CLBP-group based on LBP location

	Bilateral LBP (N= 53)		Central LBP (N= 62)		Left LBP (N= 16)		Right LBP (N= 19)		p
	M	SE	M	SE	M	SE	M	SE	
**Accuracy (%)**									
Left side									
Simple	89.6	1.6	87.4	1.5	93.4	2.9	80.5	2.7	0.01*
Complex	64.8	2.2	64.4	2.0	66.9	3.9	55.8	3.7	0.13
Right side									
Simple	91.2	1.4	87.7	1.3	89.1	2.5	85.3	2.3	0.13
Complex	65.4	2.2	64.8	2.0	63.8	4.0	60.0	3.7	0.64
**Reaction Time (s)**									
Left side									
Simple	1.67	0.08	1.80	0.07	1.76	0.14	1.87	0.14	0.51
Complex	2.81	0.12	2.92	0.11	3.03	0.22	3.14	0.20	0.51
Right side									
Simple	1.72	0.09	1.77	0.08	1.76	0.16	2.10	0.15	0.21
Complex	2.80	0.12	2.86	0.11	2.96	0.22	3.14	0.20	0.54

*Right LBP group significantly less accurate compared to the Left and Bilateral LBP group

LBP= low back pain
