# Supplementary material for: Trunk Laterality Judgement in Chronic Low Back Pain: Influence of Low Back Pain History, Task Complexity, and Clinical Correlates
Source: J Clin Med. 2025 Jul 28;14(15):5328. doi: 10.3390/jcm14155328 (PMC12347674; doi:10.3390/jcm14155328)
Supplement: Supplementary file 1 [file jcm-14-05328-s001.zip › Table S3.pdf]

Supplementary Table S3. Raw means for LRDT performance in the different participant groups

	CLBP (N=150)		Pain-free								
			PF-total (N=150)			PF-noLBP (N=107)			PF-LBP (N=43)		
	M	SD	M	SD	ES	M	SD	ES	M	SD	ES
Accuracy (%)											
Simple	88.3	9.9	92.2	6.7	0.46**	92.6	6.1	0.50**	91.0	8.1	0.28
Complex	63.9	14.2	68.5	14.4	0.32**	70.2	14.7	0.47**	64.3	12.7	0.03
Reaction time (s)											
Simple	1.76	0.54	1.54	0.56	0.40*	1.47	0.51	0.55*	1.72	0.65	0.07
Complex	2.90	0.75	2.72	0.71	0.25*	2.68	0.74	0.30*	2.79	0.65	0.15

ES= Cohen's d effect size based on raw means and standard deviation. Effect sizes are calculated between the CLBP and respective PF groups.

CLBP= chronic low back pain; PF= Pain-free; PF-LBP= Pain-free persons with a history of previous LBP; PF-noLBP= pain-free persons without a history of previous LBP; PF-total= total group of pain-free persons.

*difference with CLBP group: $p< 0.01$; **difference with CLBP group: $p< 0.001$
